# Posterior reversible encephalopathy syndrome as the first manifestation of mixed connective tissue disorder: a case report

**DOI:** 10.1186/s13256-021-02678-9

**Published:** 2021-02-02

**Authors:** Phani Krishna Machiraju, Neetu Mariam Alex, Safinaaz, Sriram Sankaran

**Affiliations:** 1 Department of General Medicine, Apollo Main Hospitals, Greams Road, Chennai, Tamil Nadu 600006 India; 2 Department of Rheumatology, Apollo Main Hospitals, Greams Road, Chennai, Tamil Nadu 600006 India; 3 Nellore, Andhra Pradesh 524002 India

**Keywords:** Atypical PRES, MCTD, Mixed connective tissue disorder, Autoimmune diseases

## Abstract

**Background:**

Posterior reversible encephalopathy syndrome (PRES) is a neurological syndrome characterised by a range of neurological symptoms and signs, and distinctive neuroimaging findings reflecting vasogenic oedema. Posterior reversible encephalopathy syndrome has been described in association with many autoimmune diseases, but its association with mixed connective tissue disorder (MCTD) is very rare. After an extensive review of the literature, we found only three cases of posterior reversible encephalopathy syndrome in association with mixed connective tissue disorder. But unlike other cases, in our patient, PRES is the presenting manifestation of mixed connective tissue disorder which is first of its kind.

**Case presentation:**

We present a 30-year-old female from Southern India who had initially reported with complaints of fever, multiple episodes of vomiting and cough with expectoration. She had accelerated hypertension and moderate thrombocytopenia. Two days later, she developed sudden onset of visual disturbances and had a drop in sensorium. Neuroimaging done was suggestive of atypical posterior reversible encephalopathy syndrome, and autoimmune workup was positive for mixed connective tissue disorder. With prompt blood pressure control and anti-seizure medications, she recovered completely.

**Conclusion:**

Early diagnosis and prompt control of blood pressure, along with anti-seizure measures, play a crucial role in management. Awareness about this rare association is essential for early diagnosis and treatment, and therefore reducing the risk of permanent neurologic deficits. This case is being reported because of its rarity.

## Background

Posterior reversible encephalopathy syndrome (PRES) is defined by a range of neurological symptoms of acute onset, focal vasogenic oedema on neuroimaging and reversibility of clinical and radiological findings [1]. Its causes are diverse, but common precipitants include acute elevations of blood pressure, renal decompensation, fluid retention, and treatment with immunosuppressive drugs [2]. The symptoms of PRES evolve rapidly over hours to days. Early diagnosis and prompt control of blood pressure along with anti-seizure measures play a key role in the management and outcome of the case, by preventing further brain damage. PRES has been described in association with many autoimmune diseases, but its association with mixed connective tissue disorder (MCTD) is very rare. In this report, we describe a case of PRES in a young female who was subsequently diagnosed to have MCTD.

## Case presentation

**Fig. 1:**
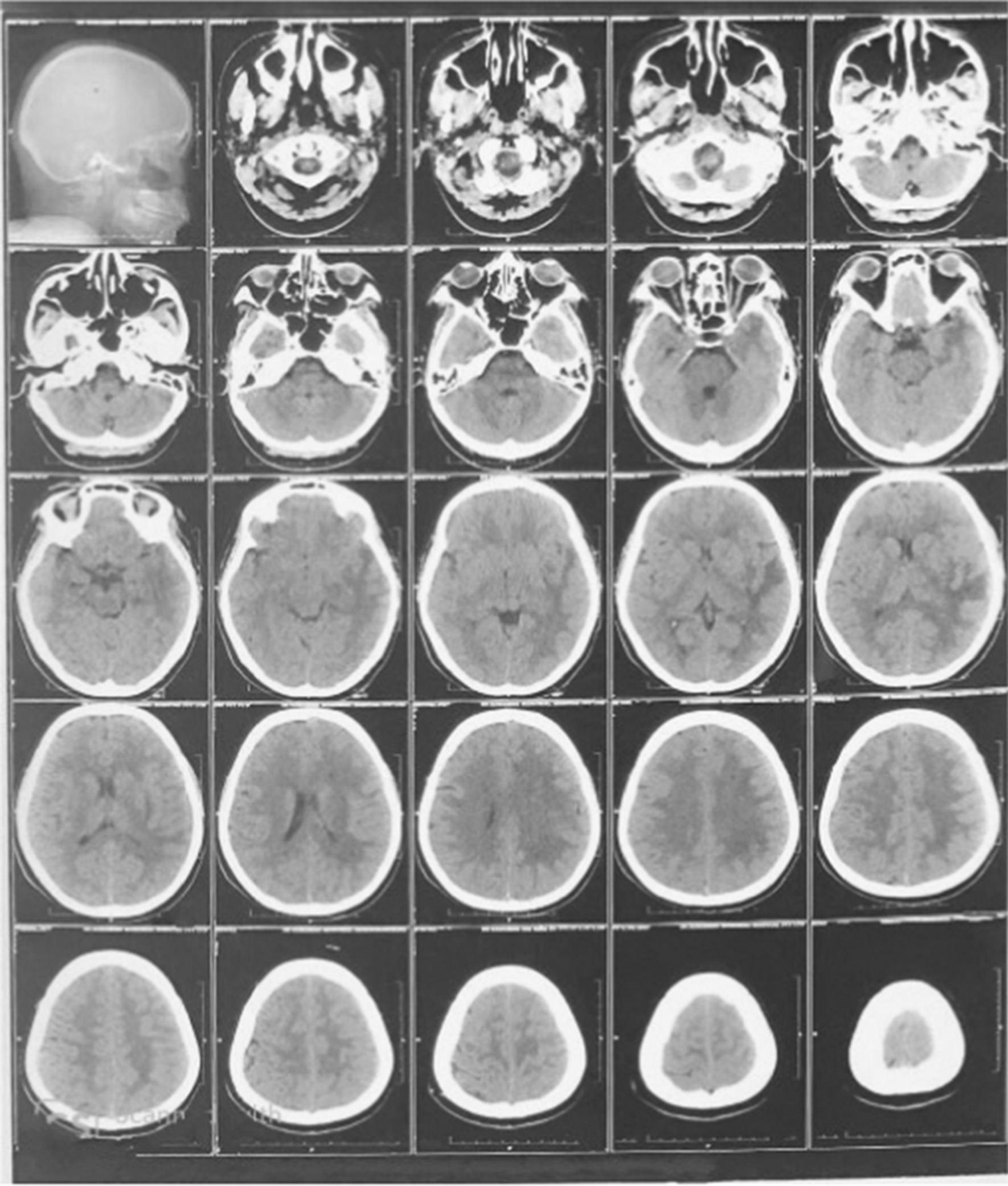
Computed Tomography (CT) scan. Brain showing ill-defined areas of parenchymal Edema in left parieto temporal lobe

Our patient was a 30-year-old female from Southern India who had initially reported to a local general practitioner with complaints of fever, multiple episodes of vomiting and cough with expectoration for 5 days. She was treated with oral antibiotics (amoxicillin/clavulanic acid 625 mg oral twice daily), but she continued to have fever spikes (mild to moderate grade with Tmax of 102.6℉). On admission and evaluation in a local hospital, she was diagnosed to have accelerated hypertension (200/110 mm of hg), moderate thrombocytopenia (60,000/mm^3^). While she was being managed with intravenous fluids and antihypertensive medications (labetalol 2 mg/min intravenous infusion), she developed sudden onset of visual disturbances and had a drop in sensorium. Computed Tomography scan brain revealed ill-defined areas of parenchymal oedema in left parieto-temporal lobe white matter, left ganglio-capsular and left hippocampal regions (Fig. 1).

**Fig. 2:**
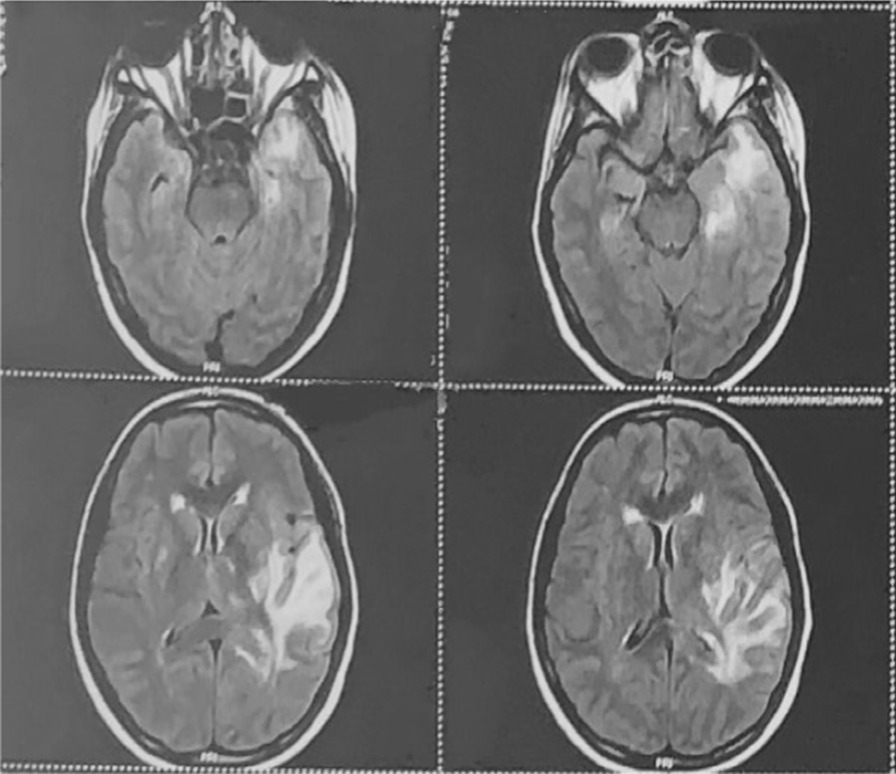
Magnetic resonance imaging (MRI). Brain with features suggestive of Atypical PRES

**Fig. 3:**
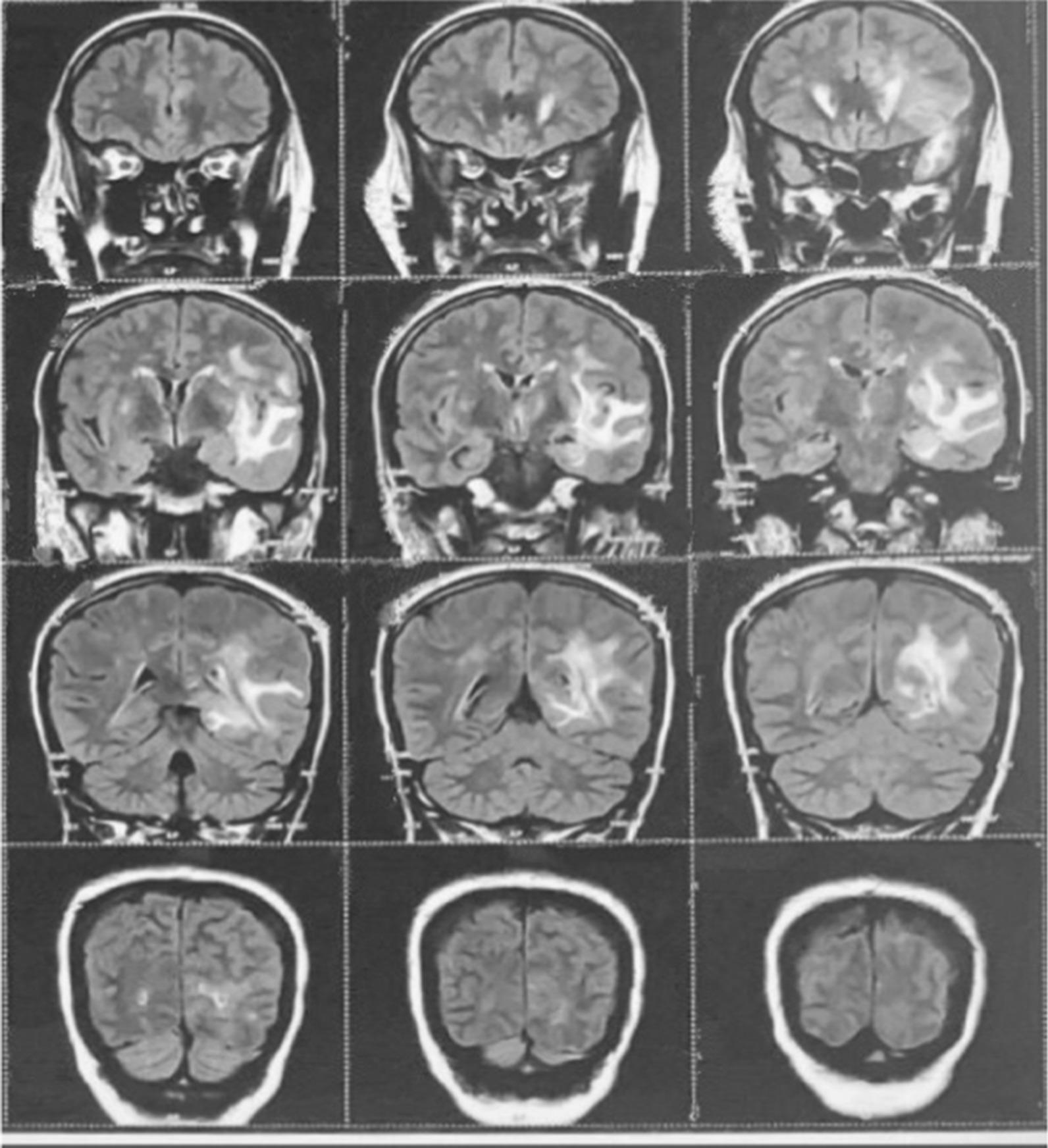
Magnetic resonance imaging (MRI). Brain with features suggestive of Atypical PRES

**Fig. 4:**
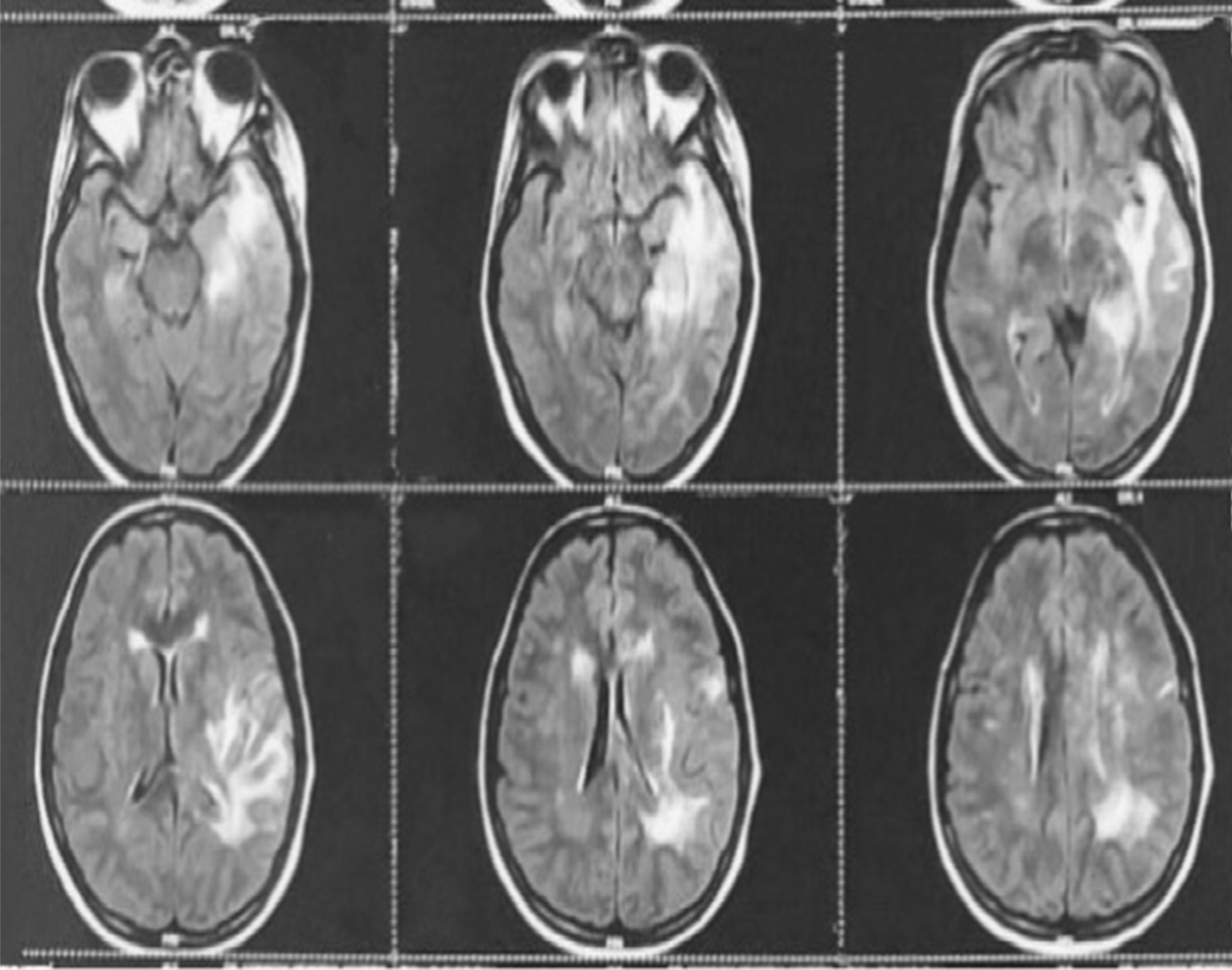
Magnetic resonance imaging (MRI). Brain with features suggestive of Atypical PRES

Magnetic resonance imaging brain (Figs. 2, 3, 4) revealed ill-defined T2/ FLAIR hyperintense signals in left parieto temporal region, multiple small foci of restricted diffusion in the left frontoparietal, parasagittal region, multiple small patchy FLAIR hyperintense lesions were seen in bilateral frontoparietal subcortical white matter, left thalamus, bilateral basal ganglion, splenium of the corpus callosum on the left side and bilateral perioptic space widening with partial empty sella. All these features were suggestive of atypical PRES or acute demyelinating encephalomyelitis.

She was treated with anti-epileptics (levetiracetam 500 mg twice daily), antihypertensive medications (labetalol 100 mg twice daily oral) and glucocorticoids (methylprednisolone 500 mg intravenously for 1 day). She was shifted to our hospital for further management. Her past medical history was notable for an aborted twin pregnancy and gestational hypertension about three years ago. She denied a history of any other co-morbidity. Her family history and psychosocial history was unremarkable. Physical examination was notable for pallor, tachycardia, and elevated blood pressure (190/100). She was drowsy but arousable with Glasgow coma scale/score of 14/15 (E3V5M6) at the time of admission. Neurological examination was notable for bilateral extensor plantar response. Cranial nerves, motor, sensory and other deep tendon reflexes examination were normal. Baseline blood investigations were notable for anaemia (Haemoglobin—8.2 g/dl), moderate thrombocytopenia (platelet count—51,000/mm^3^), and mild hypokalemia (serum K+—3.1 meq/l).

**Fig. 5:**
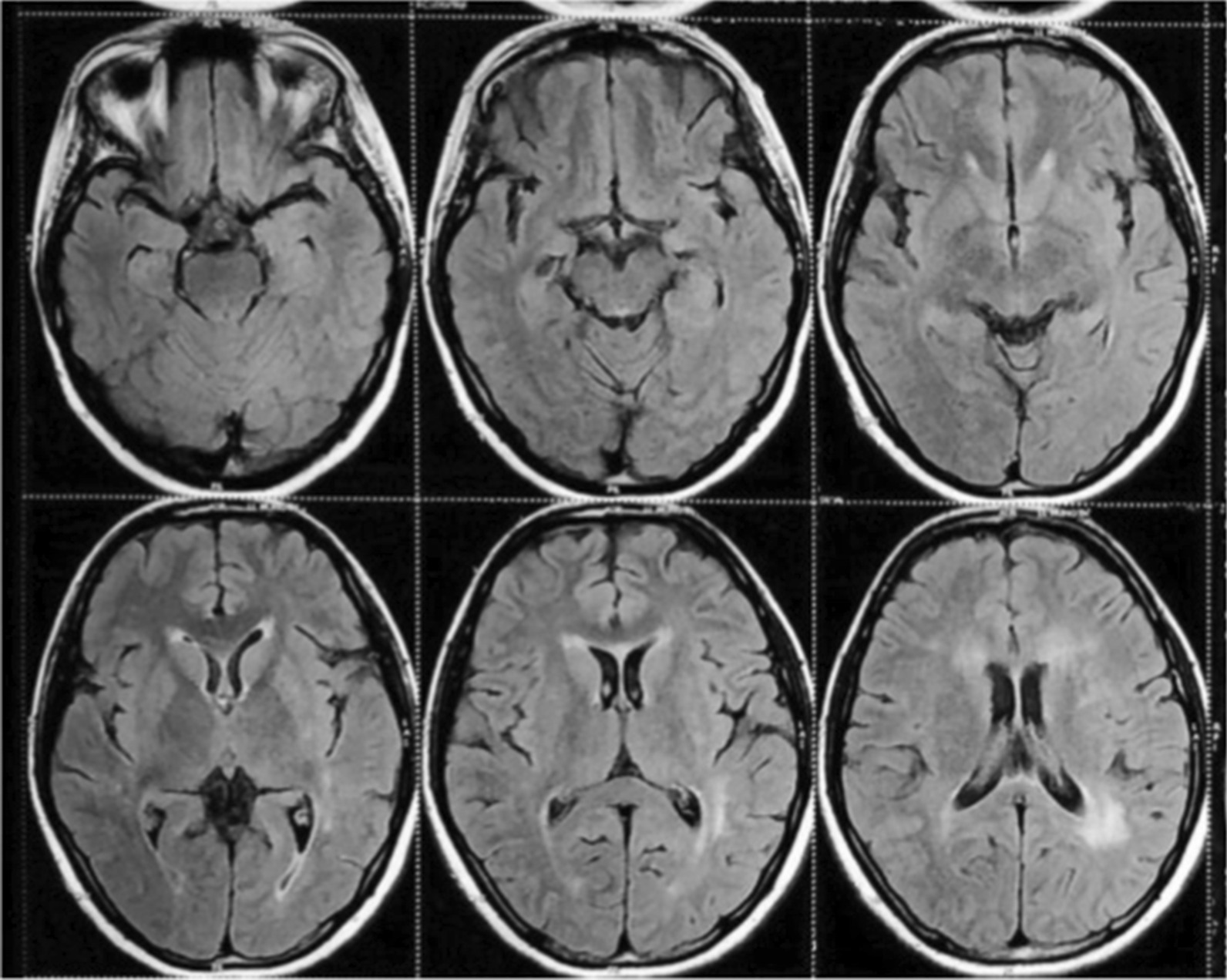
Follow-up MRI brain showing. Resolution of oedema

Blood and urine cultures were sent, and she was started on empirical antibiotics (ceftriaxone 2 g twice-daily intravenous and oral doxycycline 100 mg twice daily), antihypertensives (labetalol 2 mg/min infusion followed by 100 mg twice daily oral) and anti-epileptics (levetiracetam 500 mg orally twice daily). Infective workup done was negative (Dengue, malarial parasite (quantitative buffy coat test), H1N1 polymerase chain reaction test and Scrub typhus IgM enzyme-linked immunosorbent assay test). Guarded lumbar puncture was done to rule out infective causes of encephalomyelitis after discussing with the neurologist and infectious diseases specialist. Cerebrospinal fluid analysis (CSF) was non-contributory. (CSF was colourless, clear, no red blood cells or white blood cells were seen, Glucose was 64 mg/dl, protein was 41.1 mg/dl, GeneXpert MTB/RIF assay, Herpes simplex virus 1 & 2 real time polymerase chain reaction test were negative and CSF culture did not show any significant growth). Blood and urine cultures did not show any significant growth. Antiphospholipid antibody syndrome workup was negative. Antinuclear antibody by indirect immunofluorescence assay was positive (1:40 dilution, Intensity—4+, pattern nuclear coarse speckled). Anti-double-stranded DNA was negative, and extractable nuclear antigen antibodies profile was positive for Sm/RNP and negative for Sm. She was finally diagnosed to have posterior reversible encephalopathy syndrome (PRES) with underlying mixed connective tissue disorder. She recovered completely with antihypertensives (T. Metoprolol XL 25 mg PO BD) and anti-epileptics (T. Levetiracetam 500 mg PO BD). Repeat platelet count done on Day 4 of admission was normal (2, 64,000 cells/mm^3^). She was discharged with a plan to continue antihypertensive medications (T. Metoprolol XL 25 mg oral twice daily). She was reviewed as an outpatient after 6 weeks, and she was doing well. Follow-up MRI of Brain done after six weeks as outpatient showed resolution of oedema (Fig. 5). After discussing with patient and family in detail, our plan, in this case, was strict blood pressure control, watch out for MCTD manifestations by regular follow up.

## Discussion

Posterior reversible encephalopathy syndrome (PRES) was first described by Hinchey and his colleagues as Reversible posterior leukoencephalopathy syndrome, in a series of 15 patients in 1996 [2]. PRES is characterised by neurological manifestations of acute onset headache, visual disturbances, seizures, altered sensorium; vasogenic oedema on neuroimaging mostly in the posterior regions and reversibility of clinical and imaging findings. Women are more commonly affected than men, even after excluding eclampsia as a cause. Our patient was a young female.

PRES most commonly presents as subcortical oedema in parieto-occipital areas. Other atypical regions that may be affected include deep gray nuclei, brainstem/cerebellar hemispheres, and exceptionally the spinal cord without cerebral hemispheric involvement. These findings may lead to a diagnostic dilemma, with a delay in diagnosis and reversal of the offending condition potentially leading to a poor patient outcome [3]. In our patient, subcortical oedema was seen in the frontoparietal and parieto-temporal region, which is atypical of PRES.

PRES is associated with hypertension, renal failure and eclampsia, in patients receiving immunosuppressive therapy, chemotherapy and in patients with underlying autoimmune disease [4]. Though many autoimmune disorders like systemic lupus erythematosus, polyarteritis nodosa, granulomatosis with polyangiitis, cryoglobulinemia have been described in association with PRES, its association with MCTD is very rare. Neurological involvement as such is rare in MCTD as compared with other system involvement. It more commonly manifests as trigeminal neuralgia, aseptic meningitis and headache. After an extensive review of the literature, we found only three case reports till now of this rare association, as described in Table 1 [4–6]. All the cases reported till now are between 15 and 30 years of age.

**Table 1:** Case reports of PRES with MCTD reported till now

Case	Age	Sex	Associations/Medications	Hypertension	Clinical features	MRI findings	Outcome/sequale
Case-1	15 years	Male	Mixed connective tissue disease on cyclophosphamide pulse therapy, prednisolone and HCQ	Yes	GTCS	High signal intensities in the posterior areas of his brain.	Uncontrollable respiratory distress and eventually died
Case-2	20 years	Female	Mixed connective tissue disease	No	Headache and intermittent vomiting along with two episodes of GTCS	Bilateral hyper intense signal in occipital region on T2 weighted MRI and FLAIR, and bilateral hypo intense signal in T1 weighted MRI	Completely recovered. Follow up MRI Brain was normal
Case-3	12 years	Male	Mixed connective tissue disease, secondary membranous glomerulopathy	No	GTCS Aphasia Fever Generalized body swelling	Hyper intense signal in the parieto-occipital regions	Completely recovered
Our patient	30years	Female	Mixed connective tissue disease	Yes	Headache Fever Visual disturbances Sudden drop in sensorium	Hyperintense signals in fronto parietal and parieto temporal region	Completely recovered

Possible pathological mechanisms that could result in PRES with the background of MCTD include endothelial cell damage resulting from different autoantibodies in MCTD, vasculopathy and autonomic dysfunction [5].

Treatment for PRES is mainly aimed at blood pressure control, anti-seizure measures and other supportive measures. Blood pressure should be managed with easily titratable medications like intravenous labetalol, nitroprusside and nicardipine etc. The rapid decrease in blood pressure could cause cerebral ischemia, which is why a goal of mean blood pressure between 105 and 125 mmHg is suggested, without exceeding 25% of this reduction in the first hour [6]. As with other conditions, blood pressure fluctuations should be avoided, and the continuous administration of antihypertensive drugs under hemodynamic monitoring should be considered [1]. In our patient, we managed her blood pressure with parenteral labetalol followed by oral medications and also with anti-epileptic mediations. PRES is the first presentation of MCTD in our patient, unlike the other reported cases till now.

## Conclusion

Cases of PRES have been increasingly reported off late. It has been described to be associated with various etiopathological factors, including autoimmune conditions, but its association with MCTD is very rare. Treatment depends mainly on the underlying pathology and outcome depends on early diagnosis and treating the underlying condition. Awareness about this rare association is crucial for early diagnosis and treatment, and therefore reducing the risk of permanent neurologic deficits. Autoimmune disorders like MCTD should be considered as one of the differential diagnosis in a young female presenting with accelerated hypertension and acute onset neurological symptoms suggestive of posterior reversible encephalopathy syndrome.

## Data Availability

Not applicable.

## Abbreviations

MCTD: Mixed connective tissue disease
PRES: Posterior reversible encephalopathy syndrome
CSF: Cerebrospinal fluid
